# Trace Determination of Grouper Nervous Necrosis Virus in Contaminated Larvae and Pond Water Samples Using Label-Free Fiber Optic Nanoplasmonic Biosensor

**DOI:** 10.3390/bios12100907

**Published:** 2022-10-21

**Authors:** Yuan-Yu Chen, Chih-Lu Wu, Chia-Wei Hsu, Chih-Hui Wang, Chung-Rui Su, Chun-Jen Huang, Hau-Ren Chen, Lai-Kwan Chau, Shau-Chun Wang

**Affiliations:** 1 Department of Chemistry and Biochemistry, National Chung Cheng University, Chia-Yi 621, Taiwan; 2 Center for Nano Bio-Detection, National Chung Cheng University, Chia-Yi 621, Taiwan; 3 Department of Biomedical Sciences, National Chung Cheng University, Chia-Yi 621, Taiwan; 4 Department of Chemical and Materials Engineering, NCU-Covestro Research Center, National Central University, Taoyuan 32001, Taiwan

**Keywords:** fiber optic particle plasmon resonance biosensor, nervous necrosis virus, gold nanoparticles, larvae homogenate, pond water, on-site testing

## Abstract

We developed a fast (<20 min), label-free fiber optic particle plasmon resonance (FOPPR) immunosensing method to detect nervous necrosis virus (NNV), which often infects high-value economic aquatic species, such as grouper. Using spiked NNV particles in a phosphate buffer as samples, the standard calibration curve obtained was linear (R^2^ = 0.99) and the limit of detection (LOD) achieved was 2.75 × 10^4^ TCID_50_/mL, which is superior to that obtained using enzyme-linked immunosorbent assay (ELISA). By using an enhancement method called fiber optic nanogold-linked immunosorbent assay (FONLISA), the LOD can be further improved to <1 TCID_50_/mL, which is comparable to that found by the conventional qPCR method. Employing the larvae homogenate samples of NNV-infected grouper, the results obtained by the FOPPR biosensor agree with those obtained by the quantitative polymerase chain reaction (qPCR) method. We also examined pond water samples from an infected container in an indoor aquaculture facility. The lowest detectable level of NNV coat protein was found to be 0.17 μg/mL, which is one order lower than the LOD reported by ELISA. Therefore, we demonstrated the potential of the FOPPR biosensor as an outbreak surveillance tool, which is able to give warning indication even when the trend of larvae death toll increment is still not clear.

## 1. Introduction

Although seafood has become an important diary protein source, over-capturing has resulted in the rapid decline in the abundance of aquatic species, according to a recent report published by the Food and Agriculture Organization (FAO). In addition, unlike animal husbandry, which often exhibits a low feed conversion ratio, raising aquaculture products by fish farming benefits from a higher feed conversion ratio. Therefore, the growth of the global aquaculture industry has tremendously increased in the past decades. In 2018, the value of aquaculture production reached 82.1 million tons, which is close to the value of 96.4 tons reached by capture production. Because of this increasing trend, aquaculture production is expected to surpass capture production in 2022 [1].

On the other hand, disease outbreak severely damages regional aquaculture industry from time to time. The loss takes at least a few years from which to recover. To maintain the sustainability of the fish farming industry, the employment of reliable and easy-to-use bioanalytical tools to monitor the pathogen levels in the aquaculture environment is crucial for risk surveillance purposes. There remains a need to develop feasible methods to determine pathogens below trace level at the fish pond side prior to disease outbreak.

Nervous necrosis virus (NNV) is a type of betanodavirus, often infecting more than 50 species of fish, including grouper, causing severe neuron damage [2]. In the larvae rearing stage, the mortality of infected groupers is nearly 100% [3]. Typically when infected, grouper larvae present symptoms in 7 days and soon die within 2–4 weeks [4]. NNV is a positive-sense single-stranded RNA (ss(+)RNA) virus, which contains two nucleic acid molecules: RNA1 and RNA2. RNA1 encodes a RNA-dependent RNA polymerase and RNA2 encodes a coat protein (M.W. ~42 kDa) [5]. The viral particle of NNV, of which the size is 25–30 nm in diameter, is non-enveloped. It is in the form of an icosahedron shape with T = 3 symmetry, constructed with 180 coat proteins [6].

While conventional enzyme-linked immunosorbent assay (ELISA) methods provide adequate limit of detection (LOD) for determining NNV coat proteins in brain homogenate samples of infected fish containing more than 3 µg/mL coat proteins [7], this LOD is not capable of detecting NNV in slightly contaminated aquaculture water at the viral titer level of 10^3.5^ TCID_50_/mL, which is estimated to contain about 0.1 µg/mL crude proteins [7] and is equivalent to 10^6^ NNV copies/mL [8]. Besides, performing ELISA on site is difficult, limiting its applications in resource-limited areas. In addition, the tedious ELISA procedures rely on skilled operators to reduce false positive and false negative rates. On the other hand, the testing results of easy-to-use lateral flow assays are only semi-quantitative [9], although the digital camera-assisted visual densitometric detection method has been employed [10].

Similarly, fast molecular detection methods based on isothermal nucleic acid amplification, such as loop-mediated isothermal amplification (LAMP) and recombinase polymerase amplification (RPA), do not provide accurate results because they are prone to random polymerizations of non-target genes. Sometimes, adequate primer designs are also challenging [11,12,13].

Recently, nanoplasmonic biosensors have received great attention as they are label-free, and thus, can potentially be developed into compact and highly sensitive devices. In principle, noble metal nanoparticles and the most used gold nanoparticles (AuNPs) have an extinction band known as a particle plasmon resonance (PPR) band or a localized surface plasmon resonance (LSPR) band. The biosensing sensitivity is due to the spectral change of the nanoparticle upon adsorbate-induced refractive index (RI) change of the particle’s surrounding environment. When an analyte binds with a reciprocal recognition molecule on the particle surface, plasmonic absorption will increase. Therefore, absorbance-based PPR biosensors are particularly attractive, because they only need a simple optical setup. To improve the sensitivity of the PPR biosensing approach, we have developed a real-time and label-free fiber optic particle plasmon resonance (FOPPR) sensor, which makes use of repeated total internal reflections through an optical fiber to increase the optical path, and hence, the plasmonic absorption of the AuNP layer on the fiber core surface, thus providing several advantages, including good sensitivity at pM levels, wide linear detection range over five orders, low-cost and miniaturizable instrumentation, and affordable sensor chips. Applications of the label-free FOPPR biosensor to real samples have been demonstrated. These applications include detection of antinuclear antibodies in sera [14], tumor necrosis factor-α and matrix metalloproteinases-3 in synovial fluids [15], orchid viruses in plants [16], HLA-B27 messenger RNA in blood [17], methamphetamine in urine [18], and single nucleotide polymorphismin of DNA in blood [19].

To fulfill the aforementioned unmet need, this study applied the label-free direct method of an FOPPR biosensor to illustrate its applicability in quantifying the amount of NNV in real samples of fish bodies and pond water. Having commercially available anti-NNV antibodies immobilized on the surface of the AuNP-covered optical fiber, we used either coat protein antigens or viral particles of NNV to prepare standards to develop an FOPPR biosensor for NNV. Because of the enhanced plasmon absorbance signals resulting from repeated reflections along the sensing optical fiber, ultrasensitive detection is expected.

Although the direct method in an FOPPR biosensor is highly sensitive, it is still challenging to detect the amount of a virus in concentration levels comparable to the gold standard real-time quantitative polymerase chain reaction (qPCR) method, which amplifies the number of copies of DNA. On the other hand, methods to amplify the number of copies of a protein have not yet been discovered. As a companion to the label-free direct method using FOPPR biosensor, an alternative sandwich method-based FOPPR biosensing strategy called fiber optic nanogold-linked immunosorbent assay (FONLISA), which uses a AuNP-labelled probe as the plasmonic signal enhancer, as well as the second biorecognition element, has been recently developed. By this method, AuNPs are not initially present on the fiber core surface, and thus, the background absorbance value is zero. Then, the interactions among a detection antibody-functionalized AuNP (AuNP@Ab^D^), a target analyte molecule (A), and an immobilized capture antibody molecule (Ab^C^) form a sandwich-like AuNP@Ab^D^–A–Ab^C^ complex on the unclad surface of a sensing fiber. Therefore, one analyte molecule will lead to the increase of absorbance by one AuNP. In contrast, by the direct method in an FOPPR biosensor, the binding of one analyte molecule on the AuNP surface will result in an increase of absorptivity of the AuNP, which is several orders of magnitude smaller than the absorptivity of the AuNP itself [20]. As a result, the FONLISA method improves the sensitivity by at least three orders of magnitude [21,22,23,24,25]. Using this enhancement method, we have pushed the LOD for NNV similar to the level of the gold standard qPCR method, without carrying out amplification steps. Upon development of AuNP@Ab^D^ as a biopreserved kit for long term storage, the method has a high potential for application to the point-of-use sites.

Finally, to demonstrate the feasibility of our method in an open environment without air conditioning control as a surveillance tool for NNV outbreak warning, we analyzed the pond water samples siphoned from an infected grouper container every two or three days for eleven days by FOPPR biosensor. This is our first report on applying an FOPPR biosensor for on-site detection in an aquaculture environment. Our highly sensitive FOPPR biosensor can detect trace levels of a virus prior to the outbreak of an infection that exhibits a rapid increase of death toll.

## 2. Experimental

### 2.1. Materials

The following chemicals of reagent grade were obtained from Sigma-Aldrich (St. Louis, MO, USA): 11-mercaptoundecanoic acid (MUA), 6-mercapto-1-hexanol (MCH), N-hydroxysuccinimide (NHS), 1-ethyl-3-(3-dimethylaminopropyl)carbodiimide hydrochloride (EDC), 16-mercaptohexadecanoic acid (MHA) and ethanolamine. (3-Mercaptopropyl)-trimethyoxysilane (MPTMS, 98%) and cetyltrimethylammonium bromide (CTAB) were purchased from ThermoFisher Scientific Acros Organics (Geel, Belgium). Hydrogen tetrachloroaurate trihydrate (HAuCl_4_), sodium borohydride (NaBH4), and Tween 20 were from Showa (Tokyo, Japan). 11-Aminodecyltrithoxysilane (AUTES) was acquired from ThermoFisher Scientific Alfa Aesar (Heysham, UK). Disuccinimidyl suberate (DSS) was purchased from Ark Pharm (Arlington Heights, IL, USA). Triton X-100 was from J. T. Baker. Isopropyl β-D-1-thiogalactopyranoside (IPTG) was from ZymesetBiotek Co. Ltd. (Keelung, Taiwan). Diethyl pyrocarbonate (DEPC) was from P&C Biotech, Inc. (Taichung, Taiwan). Polyclonal rabbit antibody and monoclonal mouse antibody against NNV were from Genesis Biotech Group (Mercer, NJ, USA). Ampicillin, fetal bovine serum, L-15 Medium, and cDNA synthesis reagents were obtained from Thermo Fisher Scientific (Waltham, MA, USA). Yeast extract and tryptone were purchased from Condalab (Torrejon de Ardoz, Spain) to prepare Luria–Bertani (LB) medium. Sulfobetaine thiol (SBSH) [26] and sulfobetaine silane (SBSi) [27] were synthesized as described in previous publications. All the aqueous solutions were prepared using deionized water from a Millipore (Billerica, MA, USA) Milli-Q water purification system with a resistance of 18.2 MΩ. Multimode silica optical fibers (model F-MBC) were obtained from Newport (Irvine, CA, USA) with core and cladding diameters of 400 and 430 μm, respectively.

### 2.2. Preparation of Coat Protein of NNV

NNV coat protein, expressed in bacterium BL21(DE3) strain, was prepared in-house. The preparation procedures are described as follows. The NNV coat protein was expressed in bacterium BL21(DE3) strain, in which, the vector containing 339 aa. (1017 bps) of RNA2 gene was cloned. Single colony was inoculated into LB medium (3 mL) containing ampicillin and cultured overnight at 37 °C. Next, a proper volume of overnight culture was drawn to inoculate into 500 mL LB/ampicillin medium. This culture liquid was incubated at 37 °C for about 2 to 3 h. An aliquot of culture (1 mL) was taken out as the non-induction sample. The remaining culture liquid was used for protein induction by adding the induction reagent isopropyl β-D-1-thiogalactopyranoside (IPTG) up to 1 mM. Incubation was continued at 27 °C for another 2 h. Then, the culture was centrifuged at 6000× *g* for 15 min at 4 °C. The supernatant in the centrifuge tube was discarded. The collected pellet fraction was re-suspended in pre-chilled phosphate buffered saline-Triton (PBST) solution (25 mL), which contained 137 mM NaCl, 2.7 mM KCl, 10 mM Na_2_HPO_4_, 2 mM KH_2_PO_4_, and 1% Triton X-100. The pellet fraction was lysed with a French Press. The cell lysate was centrifuged at 4 °C, 15,000× *g* for 30 min. The supernatant (minor fraction for protein expression) in the centrifuge tube was transferred to a new tube. The pellet (major fraction for protein expression) remaining in the tube was re-suspended in 25 mL pre-chilled PBST for further purification. The protein fractions of the supernatant and pellet were analyzed by SDS-PAGE method using Coomassie Blue staining protocol.

### 2.3. Viral Particle Cultivation and Quantitation

Grouper fin cells (GF-1) were cultured at 28 °C in L-15 medium supplemented with 5% fetal bovine serum (FBS) up to 70–80% confluence. Then, viral particles of NNV isolated from naturally infected groupers (*Epinephelus lanceolatus*) were diluted with L15 medium lacking 5% FBS to 10^5^ fold and seeded into the cell culture plate to incubate at 28 °C for another 30 min. When the infectious fluid was removed, the culture plate was washed with 1× PBS solution twice, then was allowed to continue incubation at 28 °C for 7 days. Finally, the cells were collected by a cell scraper into a 15 mL tube. The collection tube was centrifuged under 1000× *g* for 5 min. Then, the supernatant was filtered through a 0.22 μm filter and stored at −80 °C in a frozen tube.

To quantify the viral content level of the cultivated solution, GF-1 cells of 2 × 10^4^ cells/well were seeded into each well of a 48-well plate containing L-15 medium plus 5% FBS and cultured overnight at 28 °C. Next, the cells were visualized under a light microscope to confirm that they were evenly distributed with over 80% confluence. Ten-fold serial dilution of the original viral sample was performed. Viral samples of 10^3^ to 10^9^-fold dilution (100 μL) and mock control were added into each well to infect the cells. After incubation at 28 °C for 7 days, the occurrences of cytopathic effect (CPE) were observed and microscopic images were acquired. The NNV titer of the cultivated solution at 1.4 × 10^7^ TCID_50_/mL was determined using Reed–Muench method. Without dilution, the crude protein content of the viral sample was determined to be ≈1.6 × 10^−4^ g/mL, using spectrophotometric method.

Using diethyl pyrocarbonate (DEPC)-treated water, 1 µg NNV RNA extracted from the aforementioned cultivated virus solution was mixed with cDNA synthesis reagents to react at 25 °C for 10 min, 42 °C for 60 min, 70 °C for 10 min, and chilled in an ice bath. The cDNA product of 3 μL was siphoned to perform the real time qPCR protocol. After mixing with a PCR master mix reagent, forward and reverse primer solutions, and fluorescence dyes, the cDNA solution was heated to 95 °C for 5 min, and then the temperature program cycle was repeated 33 times, as follows: 95 °C for 40 s, 56 °C for 40 s, and 72 °C for 40 s. The amplified product solution was determined with fluorimetry [28]. The synthesized virus gene (RNA2) was used as a standard for the calculation of the viral copy number. Standard solutions of RNA2 (1 ng/µL; M.W. 1.55 × 10^5^ Da) were diluted from 10^1^ to 10^10^ folds prior to amplification via the aforementioned PCR procedures to establish the standard curve indicating the relation between Ct number and dilution factor. Compared to the Ct numbers of each serial dilution solution of virus extract, we estimated the copy number of our cultivated virus solution as 9.6 × 10^8^ copies/µL. In other words, 1 TCID_50_/mL is equivalent to 6.9 × 10^4^ copies/mL or 69 copies/μL.

### 2.4. Apparatus

**Figure 1:**
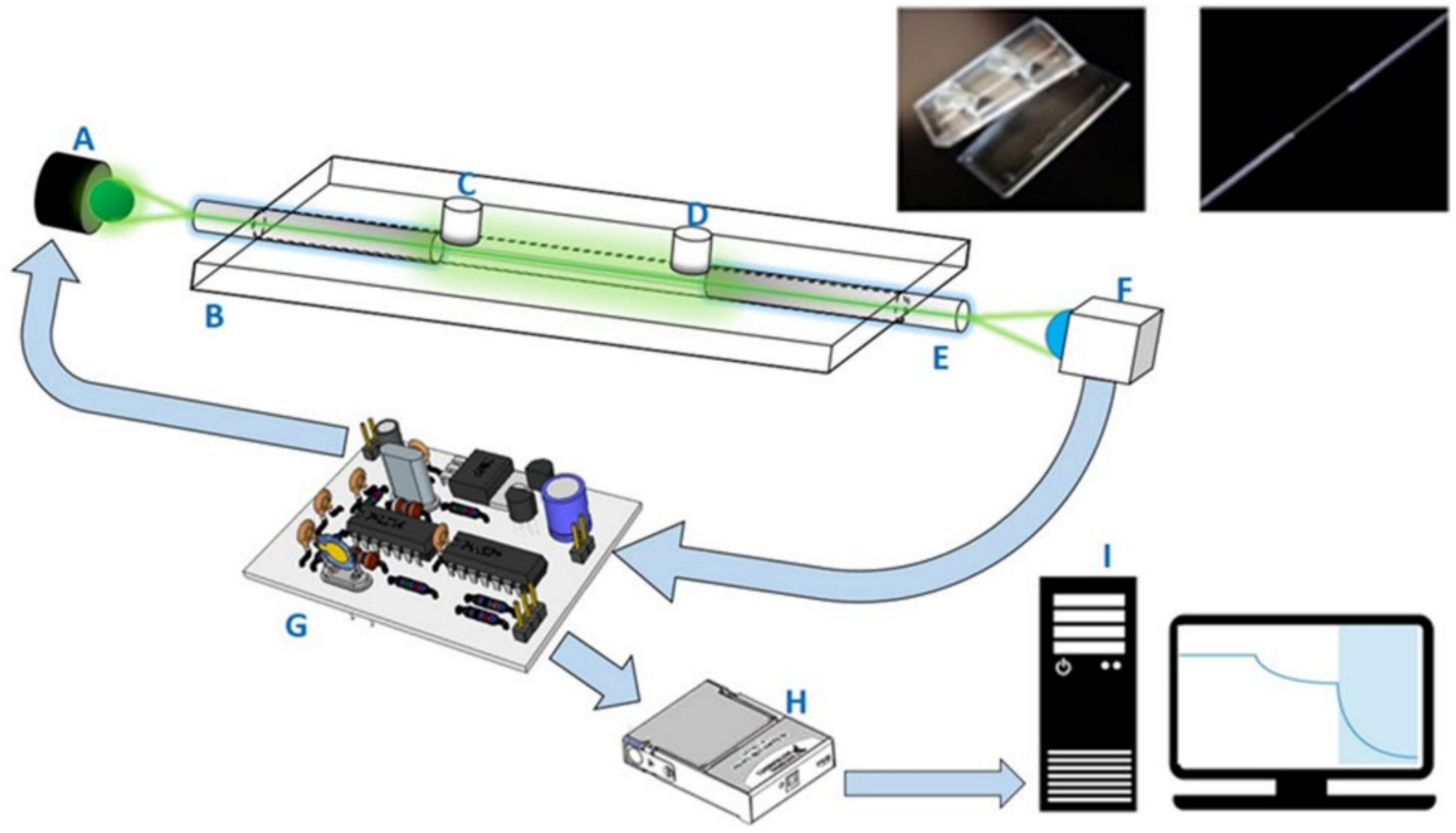
The schematic illustration of FOPPR biosensor system used in this study: A. light-emitting diode (LED); B. sensing chip; C. sample inlet port; D. sample outlet port; E. sensing fiber with one unclad segment; F. photodiode; G. circuit board with LED driver and photoreceiver amplification circuit; H. data acquisition card; I. personal computer. In the upper right, the left imbedded photograph shows the upper and lower slides of a sensor chip, while the right photograph shows the sensing fiber with one unclad segment.

The two-piece components of the sensing chips, made of cyclic olefin copolymer (COC) slides produced via an injection molding process, were similar to those in our previous publications [21,29,30]. There was a microchannel with both a depth and width of 800 μm on the bottom slide. After the bonding of the bottom and top slides, an optical fiber with an unclad segment (2 cm) serving as the sensing region was placed in the microchannel. The empty spaces at both ends were sealed. There were two holes, used as inlet and outlet ports, on the top slides to connect with the sample introduction and purging tubes. The imbedded photographs in Figure 1 shows the components of the COC sensing chip used in this study.

One desktop FOPPR biosensor (INB Kinetic) was provided by Instant NanoBiosensors Co., Ltd. (Taipei, Taiwan). The layout of the optical and electronic components of this biosensor is similar to that of our previous setup [21,29,30]. This biosensor contained one built-in circuitry to modulate a light-emitting diode (LED) light source at 1 kHz and amplification of signal from a photoreceiver, a data acquisition card, and a software loaded in a personal computer to perform lock-in amplification procedures to improve the signal-to-noise ratio of the monitored signal. A schematic illustration of this biosensor system is shown in Figure 1.

### 2.5. Preparation of Sensing Fibers for Direct Method

Spherical AuNPs were produced via the procedures described previously [31]. The mean diameter of the AuNPs was 12.3 ± 0.3 nm was determined by a transmission electron microscope (JEOL 1200 EX, Tokio, Japan). The nanoparticles were immobilized on the unclad segment, i.e., the sensing region, of an optical fiber via self-assembly. The steps to prepare the sensing fiber, including chemical modification of the sensing region surface and conjugation of immune-probe on AuNP surface, are described as follows. First, an unclad segment of an optical fiber was immersed into a toluene solution of 2% MPDMS for 4 h to produce a self-assembled monolayer (SAM) of MPDMS presenting thiol groups to react with AuNPs so that the AuNPs were immobilized on the surface. Next, MUA (2 mM) and MCH (2 mM) were mixed at a volume ratio of 1:4 in an ethanol solution. The optical fiber with AuNP-coated unclad segment was then immersed in the ethanol solution for 16 h under room temperature so that it would be covered with a mixed SAM of MUA and MCH on the AuNP surface. When the full monolayer coverage was accomplished, the fiber was finally rinsed with de-ionized water and dried with nitrogen gas. Then, the carboxylic group of MUA in the mixed SAM was activated when the unclad fiber was immersed into an aqueous solution of EDC (0.1 mM) and NHS (0.025 mM) for 1 h. Upon the activation of the carboxylic group, the segment was immersed into an antibody solution (10^−5^ g/mL) in PBS (pH 7.2) for 2 h. When antibody-AuNP conjugation was accomplished, the nonreacted carboxylic group of MUA was capped by injecting an ethanolamine solution (1 M) into the microchannel for 30 min.

### 2.6. Analysis of Samples by Direct Method

For biosensing experiments based on the direct method in the FOPPR biosensor, a phosphate-buffered saline (PBS) solution (pH 7.4) was injected into a sensing chip to thoroughly rinse the sensing region surface and to establish a baseline that shows the transmitted light intensity through the sensing fiber (I_0_). Subsequently, when a sample solution of NNV coat protein or NNV particles was filled into the microchannel of the sensing chip, the analyte (NNV coat protein or NNV particles) would dock on the sensing region surface through binding with the immune probe on AuNPs, resulting in the decrease of transmitted light intensity through the sensing fiber (I_S_). For quantitative analysis, the intensity I_S_ is normalized with respect to I_0_, and the normalized response is defined as ΔI/I_0_, where ΔI = I_0_ − I_S_, which is related to the amount of analyte docked on the sensing region surface [20]. At least three chips were prepared to replicate each measurement in determining the virus level of NNV particle sample for method validation.

### 2.7. Preparation of AuNP@Ab^D^ Conjugate and Sensing Fibers for FONLISA Detection

The procedures to prepare sensing fibers and AuNP@Ab^D^ conjugate for FONLISA are described as follows. AuNPs were first dispersed in 0.001 % (*v*/*v*) Tween 20 solution. Then a methanol solution of MHA and SBSH, of which the final concentrations were 0.02 mM and 0.08 mM, respectively, was added and incubated for at least 16 h. MHA and SBSH were chemisorbed on AuNP via thiol groups. The aggregation of AuNPs was avoided because of negative charged terminal group on the zwitterionic SBSH. Upon activation of the carboxylic group on MHA using EDC/NHS procedures, a PBS solution (pH 7.2) containing the detection antibody (~10^−5^ g/mL) was added and allowed to react for 16 h to conjugate the detection antibody on AuNP surface. The non-activated MHA sites were deactivated by reacting with a solution of 1 M ethanolamine in water at a pH of 8.5 for 30 min.

The sensing fiber for FONLISA was prepared stepwise, as follows. First, the unclad segment of an optical fiber was immersed into a solution of 5 mM AUTES and 10 mM SBSi in absolute alcohol for 16 h to assemble a mixed SAM on the fiber core surface. Next, the AUTES/SBSi-modified unclad segment was then immersed in an 80 mM DSS solution in ethanol for 16 h under room temperature to activate the amine group on AUTES. When the activation of amine group was accomplished, the fiber was rinsed with deionized water and dried with nitrogen gas. Finally, the segment was immersed in a PBS solution (pH 7.2) containing the capture antibody (~10^−5^ g/mL) for 2 h to conjugate the detection antibody on the sensing region surface. The non-activated AUTES sites were deactivated by reacting with a solution of 1 M ethanolamine in water at a pH of 8.5 for 30 min.

In this case, the sensing region surface was covered with a mixed self-assembled monolayer (SAM) containing a zwitterion component to avoid non-specific binding of AuNP@Ab^D^ conjugate on sensing region surface. Then the capture antibody was conjugated on the sensing region surface. For biosensing experiments based on FONLISA, similar procedures as the direct method were used except that the sample solution was first mixed with a solution of AuNP@Ab^D^ conjugate at a fixed concentration in a 1:1 volume ratio and incubated for 15 min prior to filling into the microchannel to obtain enhanced signals.

### 2.8. Determination of Real Samples

One larvae of infected grouper of 15 g was blended in PBS buffer and diluted to 150 g. One aliquot of 1 mL homogenate solution was siphoned to determine the NNV content. First, the homogenate solution was diluted using PBS buffer to ten folds prior to running through a filtration membrane of which the pore size is 0.2 μm. The filtrate solution was then diluted serially to 2, 4, 8, 16, and 32 folds prior to loading into a sensor chip to ensure the detected signal intensities of the diluted filtrate samples were within the dynamic range of the standard curve using the virus particle solutions. In the direct FOPPR biosensor method, the following procedure was used to correct the refractive index (RI) difference between the sample and the blank (i.e., PBS solution). When a sample sensorgram reached steady state, a PBS solution was injected to flush the previously loaded solution and then this transmitted light intensity through the sensor fiber was taken as I_S_ in order to correct the over-estimated absorbance caused by the matrix effect.

One aliquot (800 µL) of infected grouper homogenate in PBS was added with lysis reagent to dissolve tissue. Repeating washing and centrifugation steps using chloroform, isopropanol, and ethanol, were performed. Collected pellets were dissolved in DEPC treated water to extract RNA, determined via the procedures to quantify the cultivated NNV solution.

Pond water samples of 250 mL were collected every two or three days from an aquaculture container of about 1000 pieces of grouper larvae at the beginning of the culturing process. The container was an in indoor facility belonging to a local fish farmer. Whenever suspected infected groupers by NNV arose because of contaminated feeds, an aliquot of 2.0 mL was withdrawn from each sample to run through a filter paper by gravity for clean-up. A filtrate solution of 100 μL was loaded into a sensor chip to obtain a sensorgram.

Because of high salinity (~10‰), the RI of clean pond water is higher than that of PBS solution. To correct the RI difference between the sample and PBS solution, when a sample sensorgram reached steady state, a PBS solution was injected to flush the previously loaded solution, and then this transmitted light intensity through the sensor fiber was taken as I_S_ in order to correct the over-estimated absorbance caused by the matrix effect.

## 3. Results and Discussion

### 3.1. Construction of Biosensors

**Scheme 1:**
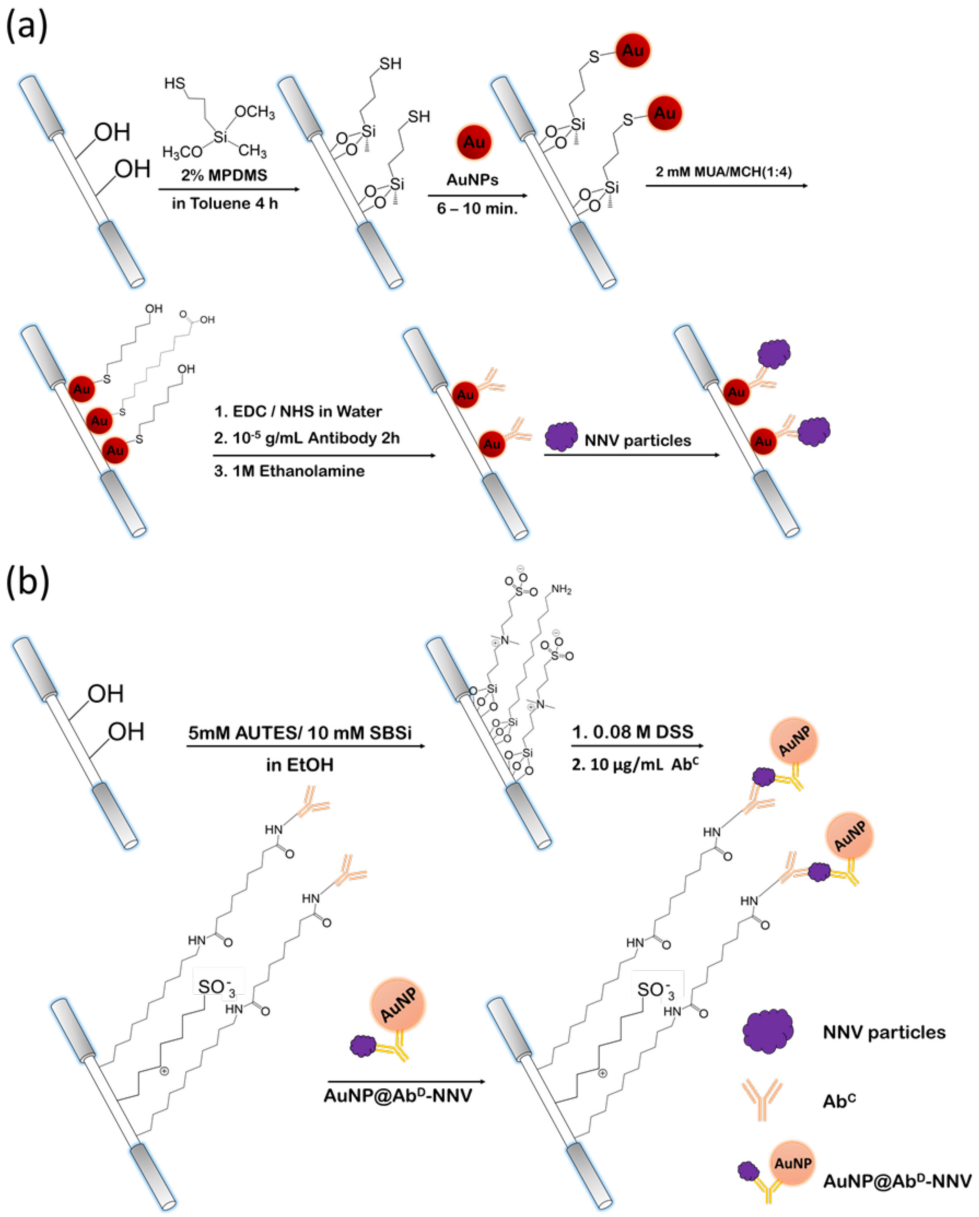
Schematic of the steps involved in the fabrication of sensing fibers and the corresponding biosensing strategies for (**a**) direct method and (**b**) FONLISA method.

Scheme 1a shows a schematic illustration of the label-free direct method of the FOPPR biosensor for detection of NNV. A partially unclad optical fiber was modified with a thiol group by immersing in a toluene solution of 2% MPDMS. Then, the thiol-functionalized fiber was immersed in a solution of AuNPs to immobilize the AuNPs on the fiber core surface. Next, the AuNP surface was modified with a mixed self-assembled monolayer (mSAM) by immersing in a mixture consisting of MUA and MCH, where MUA provides a carboxyl (−COOH) end group and MCH provides a hydroxyl (−OH) end group. In this mSAM, the carboxyl group presents a site for bioconjugation of anti-NNV antibody while the hydrophilic hydroxyl group is used to minimize nonspecific adsorption of matrix molecules [20].

Scheme 1b shows a schematic illustration of the FONLISA method of FOPPR biosensor for detection of NNV. A partially unclad optical fiber was first modified with a mSAM by immersion in an ethanol solution of AUTES and SBSi, where AUTES provides an amine (−NH_2_) end group for bioconjugation of anti-NNV antibodies as a capture probe (Ab^C^), and SBSi provides a zwitterionic terminal end to resist nonspecific adsorption of matrix molecules and AuNP@Ab^D^ conjugation on the fiber core surface [21,26]. In the presence of an analyte (A), in this case NNV, the immobilized capture antibodies (Ab^C^) on the fiber core surface and the free AuNP@Ab^D^ conjugate will interact with NNV to form a sandwich-like AuNP@Ab^D^–A–Ab^C^ complex on the fiber core surface.

### 3.2. Detection of NNV by Label-Free Direct Method

**Figure 2:**
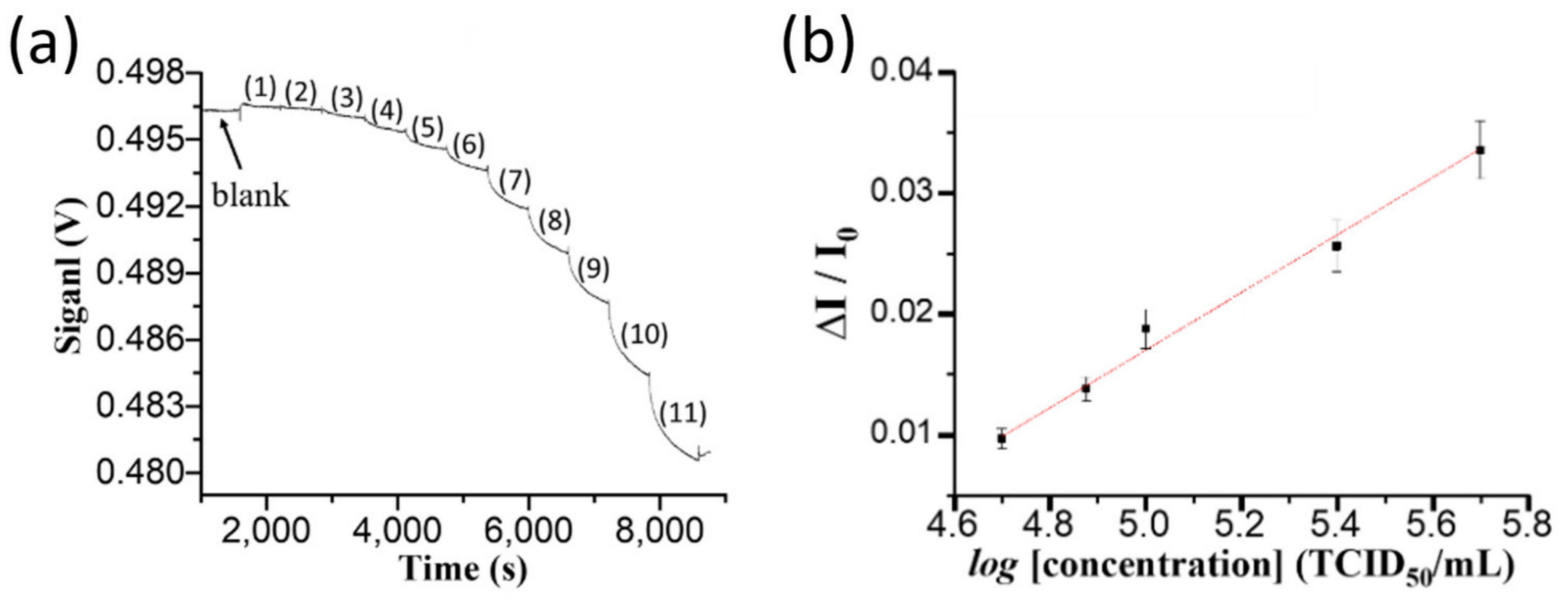
(**a**) The sensorgram obtained by sequentially injecting NNV standard solutions at concentration of (1) 1.0 × 10^3^, (2) 2.5 × 10^3^, (3) 5.0 × 10^3^, (4) 7.5 × 10^3^, (5) 1.0 × 10^4^, (6) 2.5 × 10^4^, (7) 5.0 × 10^4^, (8) 7.5 × 10^4^, (9) 1.0 × 10^5^, (10) 2.5 × 10^5^, and (11) 5.0 × 10^5^ TCID_50_/mL. (**b**) The standard calibration curve of the normalized response (ΔI/I_0_) versus the logarithm value of NNV concentration (log[C]). Each point is the mean of 3 repeated measurements.

The stock solution containing cultivated NNV particles was diluted using PBS buffer to the following concentrations, 5.0 × 10^5^, 2.5 × 10^5^, 1.0 × 10^5^, 7.5 × 10^4^, 5.0 × 10^4^, 2.5 × 10^4^, 1.0 × 10^4^, 7.5 × 10^3^, 5.0 × 10^3^, 2.5 × 10^3^, and 1.0 × 10^3^ TCID_50_/mL. These samples were injected sequentially from low to high concentration into the sensing chip to record the sensorgram, as shown in Figure 2a. Figure 2b shows that in the range of 5.0 × 10^4^ to 5.0 × 10^5^ TCID_50_/mL, of which the crude protein contents are about 8 × 10^−7^ to 8 × 10^−6^ g/mL and is equivalent to 3.4 × 10^6^ to 3.4 × 10^7^ copies/μL, the standard calibration curve of normalized response (ΔI/I_0_) versus the logarithm value of NNV level (log[C]) is linear (R^2^ = 0.99). The linear regression equation obtained from the calibration curve is (ΔI/I_0_) = 0.024 × (log[C]) − 0.10, where C is the concentration of NNV particles in TCID_50_/mL. The LOD is calculated based on the signal-to-noise ratio of 3 and is calculated to be 2.75 × 10^4^ TCID_50_/mL (i.e., 1.9 × 10^6^ copies/μL). The experimental results also exhibited good reproducibility (coefficient of variation, CV < 8.6%). The virus concentration range of this standard calibration curve is suitable to determine NNV levels in infected fish organs [32].

**Figure 3:**
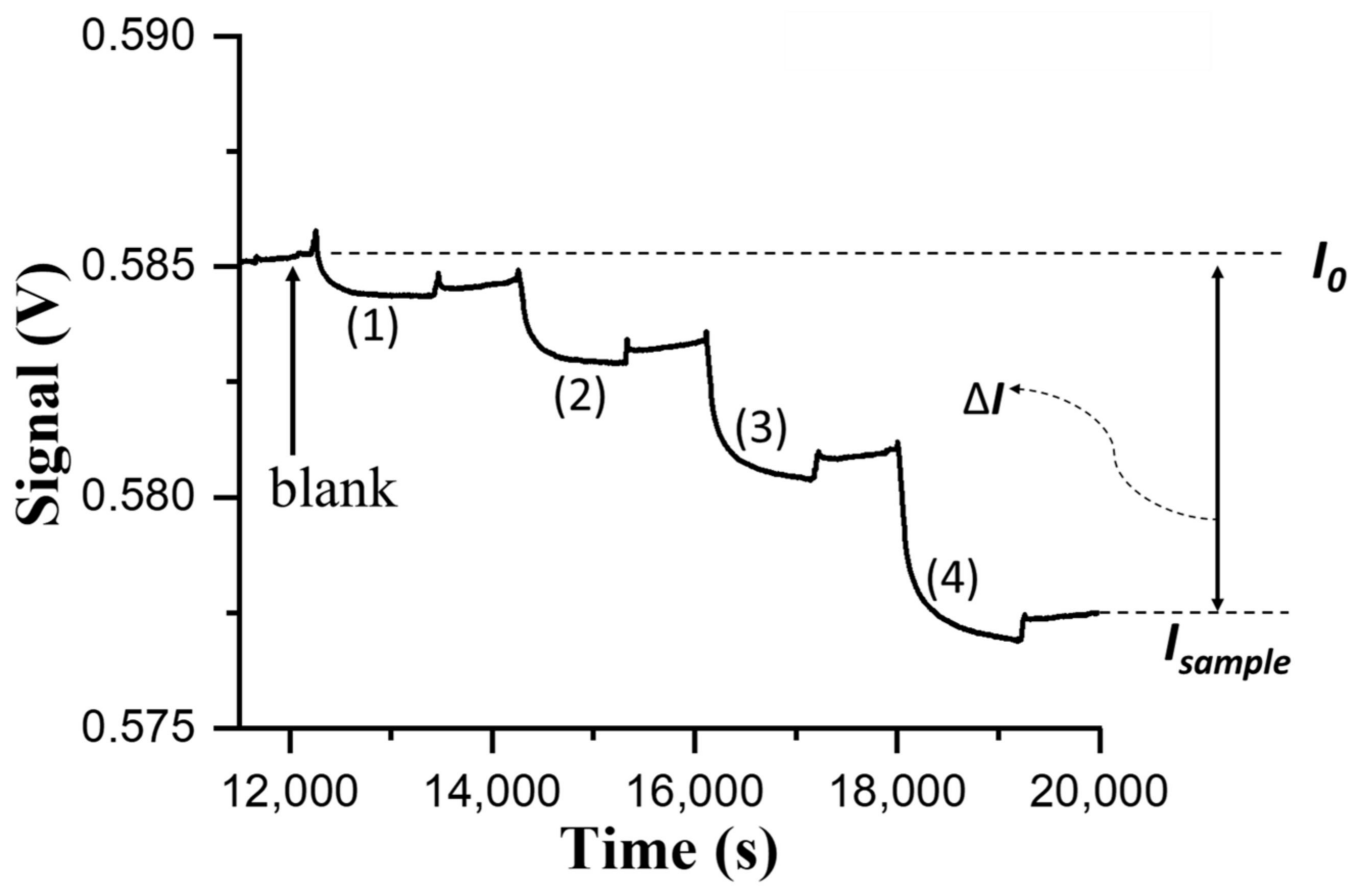
The sensorgram obtained by sequentially injecting diluted filtrate samples of NNV-infected larvae homogenate with dilution factor of (1) 320, (2) 160, (3) 80, and (4) 40.

When a sample of the larvae homogenate of 10-fold dilution was filtered, the serially diluted filtrate solutions from one fold to 32 folds (i.e., totally 10-fold to 320-fold) were sequentially loaded into a sensing chip from high dilution ratio to low dilution ratio to record the sensorgram, as shown in Figure 3. Even though the larvae homogenate sample had been filtered, the RI of the diluted filtrate solutions are different from that of the blank (PBS). Therefore, a PBS solution was loaded to flush through the sensing chip after each sample injection to clear the diluted filtrate sample and to correct the signal intensity due to the effect of bulk RI change on the FOPPR signal [33]. The corrected signal is denoted as I_sample_ and was used to calculate ΔI. It should be noted that although the normalized responses of the diluted filtrate samples at higher dilution ratios had RI not too different from that of the blank, the normalized responses were often too small to be accurately measured. On the other hand, the normalized response (ΔI/I_0_) of a 40-fold diluted filtrate sample was about ~0.014, falling within the concentration range of the standard curve as shown in Figure 2b. Therefore, the diluted filtrate samples at 40-fold dilution ratio were used as the injection samples to determine virus content level. The determined concentration using three replicates was 5.2 × 10^6^ TCID_50_/mL (3.6 × 10^8^ copies/μL) with CV < 15%. For comparison, the concentration of the same diluted filtrate sample using quantitative PCR method was 1.0 × 10^7^ TCID_50_/mL (6.9 × 10^8^ copies/μL). The acceptable consistency between these two results indicate the feasibility of the FOPPR biosensor to detect the NNV content in grouper larvae samples.

### 3.3. Detection of NNV by FONLISA Method

**Figure 4:**
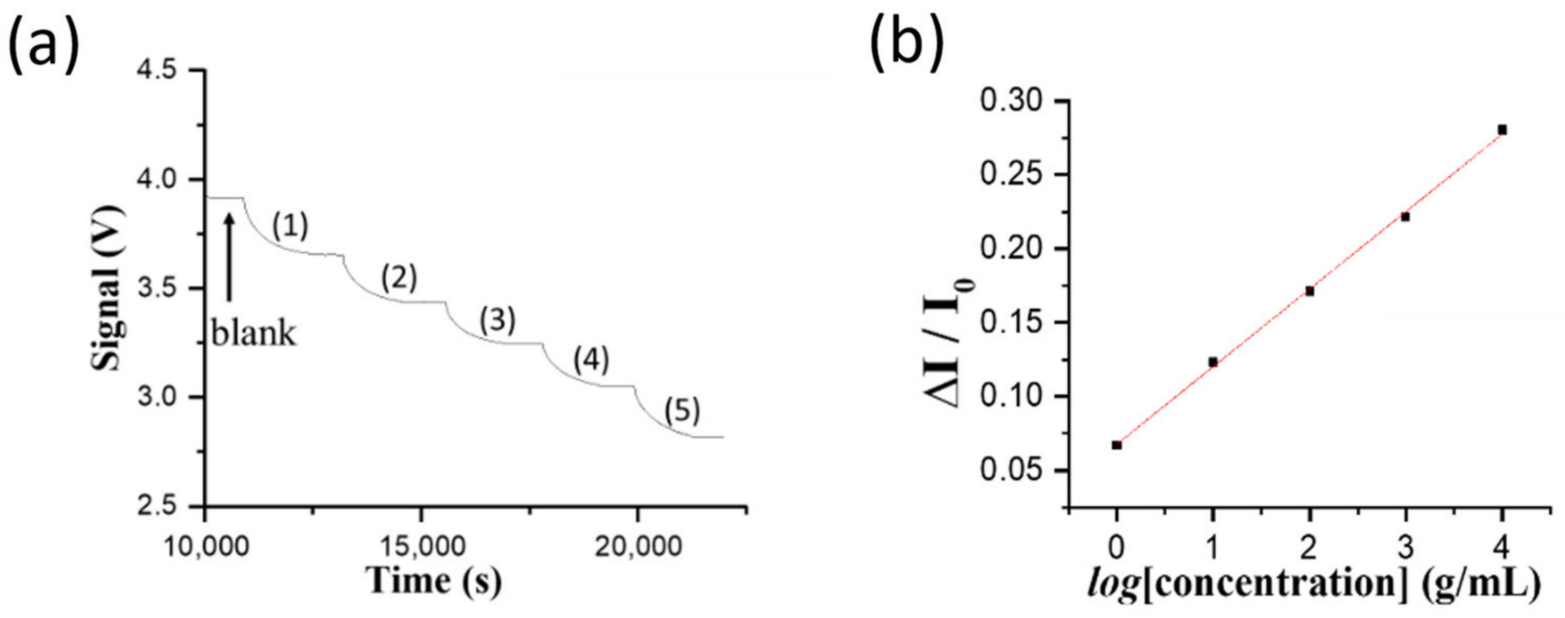
(**a**) The sensorgram obtained by sequentially injecting NNV standard solutions at concentrations of (1) 1.0 × 10^0^, (2) 1.0 × 10^1^, (3) 1.0 × 10^2^, (4) 1.0 × 10^3^, and (5) 1.0 × 10^4^ TCID_50_/mL in the presence of AuNP@Ab^D^ conjugate. (**b**) The standard calibration curve of the normalized response (ΔI/I_0_) versus the logarithm value of NNV concentration (log[C]).

Although real-time qPCR is a routinely used tool in most laboratories, it cannot differentiate between viable and nonviable viruses due to the persistence of DNA in fully or partially intact viruses [34]. Antigen tests should be considered when virus viability is being assessed and may be useful to identify NNV infected populations [35]. However, current antigen tests for NNV often lack sensitivity as compared to qPCR. Hence, we explore the use of the ultrasensitive FONLISA method for the immune detection of NNV. The stock solution containing cultivated NNV particles was diluted using PBS buffer to the following concentrations, 10,000, 1000, 100, 10, and 1 TCID_50_/mL. These samples were incubated with a fixed concentration of AuNP@Ab^D^ conjugate prior to injection into the sensing chip. As shown in Figure 4a, these samples were injected sequentially from low to high concentration into the sensing chip to record the sensorgram. Figure 4b shows the corresponding standard calibration curve of normalized response (ΔI/I_0_) versus the logarithm value of NNV level (log[C]). The plot is highly linear (R^2^ = 0.998) and the linear regression equation obtained from the calibration curve is (ΔI/I_0_) = 0.0526 × (log[C]) + 0.0677. As the lowest concentration of the diluted filtrate sample at 1 TCID_50_/mL was detectable by FONLISA and our qPCR results showed that the virus sample of 1 TCID_50_/mL was equivalent to ~70 copies per μL of NNV, the LOD of the FONLISA method for the cultivated NNV virus solution is lower than 70 copies/μL. Since the detection channel in the sensing chip contains less than 20 μL sample, a virus sample containing about 1400 virus copies should result in a clearly detectable signal by FONLISA. Such a LOD in terms of virus copies is comparable with the conventional qPCR method, which provides the LOD at 1900 virus copies [27], suggesting that the FONLISA method of the FOPPR biosensor is potentially capable of detecting viable NNV with similar sensitivity as the qPCR method.

### 3.4. On-Site Detection of NNV in Pond Water

Studies indicate that NNV particles are unstable in aqueous conditions [31]. Therefore, viral particles should hardly remain intact in pond water. On the other hand, because these partially degraded virus particles still contain coat proteins, the concentration level of NNV coat proteins in pond water may be an adequate measure of the extent of current infection. Therefore, we used the standards of coat proteins spiked in clean pond water to establish the standard calibration curve.

**Figure 5:**
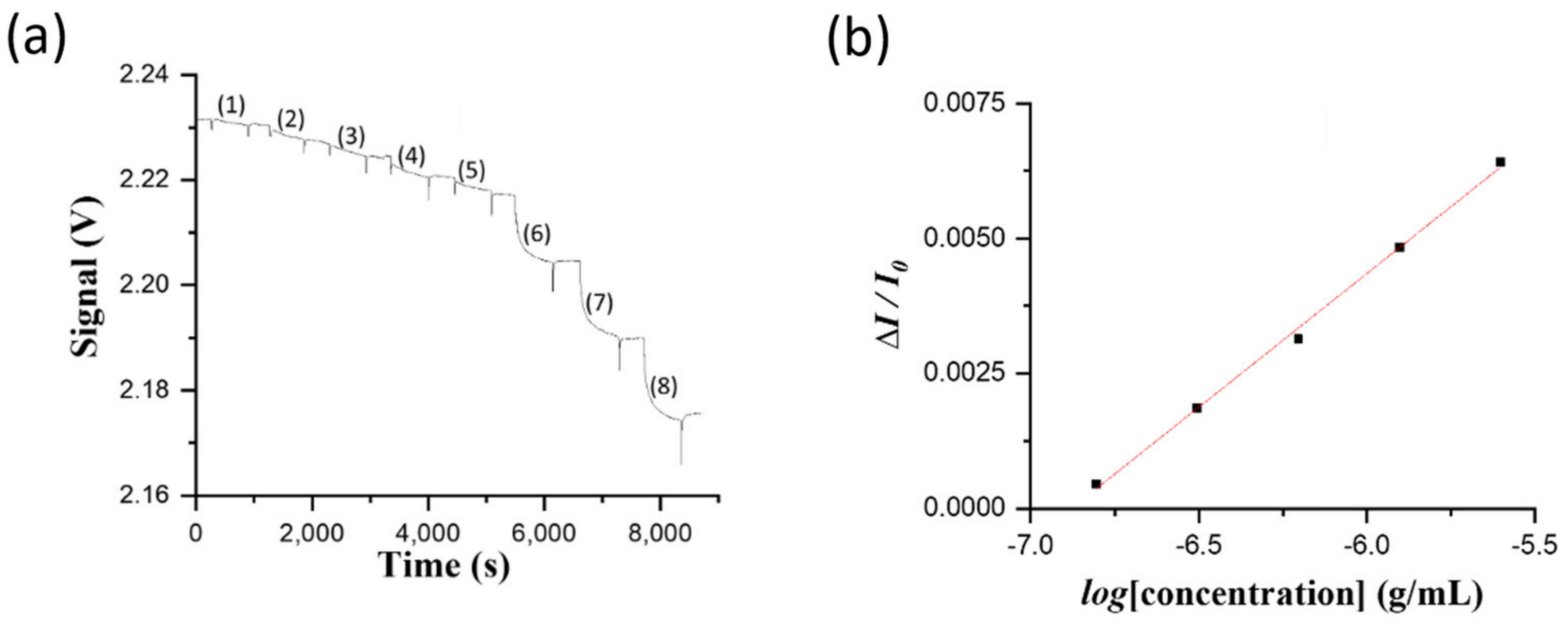
(**a**) The sensorgram obtained by sequentially injecting NNV coat protein standard solutions at concentrations of (1) 1.4 × 10^−7^, (2) 2.8 × 10^−7^, (3) 5.6 × 10^−7^, (4) 1.25 × 10^−6^, (5) 2.5 × 10^−6^, (6) 5.0 × 10^−6^, (7) 1.0 × 10^−5^, and (8) 2.0 × 10^−5^ to g/mL. (**b**) The corresponding standard calibration curve of the normalized response (ΔI/I_0_) versus the logarithm value of NNV coat protein concentration (log[C]), over the range of 1.4 × 10^−7^ to 2.5 × 10^−6^ g/mL.

Because there was no air conditioner in the indoor aquaculture facility, we finished up the assay procedures in less than three hours to avoid possible signal intensity drifting caused by the obvious change of ambient temperature over longer hours. Therefore, only one standard solution was used for each concentration without repeated measurements to establish the standard curve. The standards of NNV coat proteins were prepared at 1.4 × 10^−7^, 2.8 × 10^−7^, 5.6 × 10^−7^, 1.25 × 10^−6^, 2.5 × 10^−6^, 5.0 × 10^−6^, 1.0 × 10^−5^, and 2.0 × 10^−5^ g/mL using clean pond water. Then, they were sequentially loaded into a sensing chip from low to high concentration to obtain the sensorgram, as shown in Figure 5a. PBS solution was loaded to flush through the sensing chip after each sample injection to clear the sample and to correct signal intensity due to the bulk RI different between the blank and the sample. Figure 5b shows the standard curve of normalized response (ΔI/I_0_) versus the logarithm value of NNV level (log[C]). The plot is highly linear (R^2^ = 0.996) and the linear regression equation obtained from the calibration curve is Y = 0.00495 × (log[C]) + 0.034 over the concentration range of 1.4 × 10^−7^ to 2.5 × 10^−6^ g/mL. The LOD is calculated to be 0.14 × 10^−6^ g/mL.

**Figure 6:**
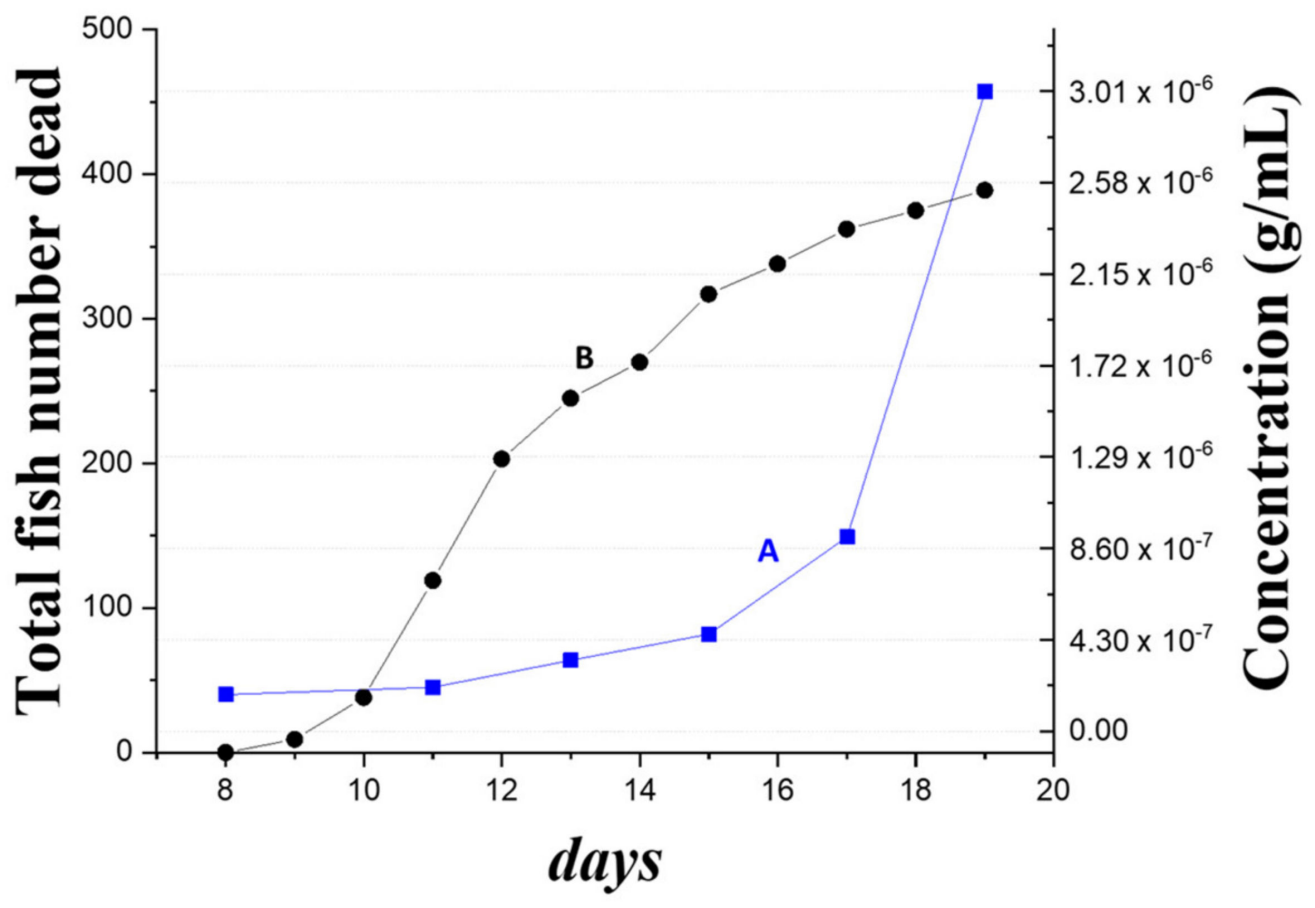
The detected concentration of NNV coat proteins in an infected pond using FOPPR biosensor (curve A) from the eighth day to the nineteenth day during a larvae cultivation process; the death toll of grouper larvae (curve B) in the same time span.

To test the feasibility of using the concentration level of NNV coat proteins in pond water to monitor the status of NNV infection, we monitored the death toll of grouper larvae and measured the concentration of NNV coat proteins in pond water over the same time span, starting on the eighth day of the larvae culturing process. In Figure 6, curve A shows the increase of detected NNV coat protein concentration in pond water with samples collected every two or three days for eleven days, i.e., between the eighth day and the nineteenth day. In the same graph, the death toll of larvae pieces are indicated in curve B. Although no fish died during the first eight days of the culturing process, NNV coat protein concentration was found to be 1.7 × 10^−7^ g/mL on the eighth day. This concentration level was one order of magnitude lower than the reported LOD using the ELISA method [7]. The detected concentration of NNV coat proteins gradually increased after the eighth day. Interestingly, dead larvae pieces were firstly found on the ninth day and then the death toll started to increase rapidly on the eleventh day, which clearly indicated the outbreak of NNV infection. Within the time span of eleven days, from the eighth day to the nineteenth day, the death toll quickly accumulated to nearly half of the original larvae population, while the NNV coat protein concentration was determined to be 3.0 × 10^−6^ g/mL on the nineteenth day, thus indicating that the pond water was highly polluted. On the other hand, from the eighth day to the fifteenth day, the qPCR results of these pond water samples were not distinguishable from the result of the control sample using clean pond water. On the seventeenth and nineteenth days, the qPCR results were found to b e 9.6 × 10^2^ copies/μL and 3.1 × 10^4^ copies/μL, respectively. Using these qPCR results, the crude protein levels of the seventeenth day and nineteenth day samples were estimated as 4.5 × 10^−9^ g/mL and 1.45 × 10^−8^ g/mL, respectively; nearly 200-fold less than the FOPPR determination results of NNV coat proteins, 8.0 × 10^−7^ g/mL and 3.0 × 10^−6^ g/mL, respectively. The comparison results suggest that the qPCR method was not suitable for quantifying NNV levels in polluted pond water because viral RNAs were not stable in aqueous condition.

The aforementioned findings show that the FOPPR biosensing method can detect the existence of NNV in pond water three days prior to the occurrence of infection outbreak. The potential feasibility of using an FOPPR biosensor as the surveillance tool in a pond site was successfully demonstrated.

## 4. Conclusions

We successfully developed the label-free fiber optic nanoplasmonic biosensing method to detect grouper NNV within 20 min. With LOD superior to that of using ELISA, we demonstrated the feasibility of detecting the larvae homogenate samples of NNV-infected grouper, and obtained agreeable results to those of the qPCR method. Besides, using the FONLISA method to enhance the sensing sensitivity without carrying out any amplification steps, we improved the LOD to <1 TCID_50_/mL, which is comparable to the published LOD of the conventional qPCR method.

We also successfully demonstrated that this easy-to-use nanoplasmonic biosensor is a suitable surveillance tool to monitor NNV levels in grouper larvae pond water in an open environment without air conditioning control. With the high sensitivity of our method, a warning indication can be revealed when the trend of larvae death toll increment is still not clear.

## Data Availability

All relevant data are available in the manuscript.
